# New Wine in an Old Bottle: Utilizing Chemical Genetics to Dissect Apical Hook Development

**DOI:** 10.3390/life12081285

**Published:** 2022-08-22

**Authors:** Yalikunjiang Aizezi, Yinpeng Xie, Hongwei Guo, Kai Jiang

**Affiliations:** 1 Institute of Plant and Food Science, Department of Biology, School of Life Sciences, Southern University of Science and Technology (SUSTech), Shenzhen 518055, China; 2 Key Laboratory of Molecular Design for Plant Cell Factory of Guangdong Higher Education Institutes, Southern University of Science and Technology, Shenzhen 518055, China

**Keywords:** chemical genetics, phytohormones, apical hook development, Auxin, Polar auxin transport

## Abstract

The apical hook is formed by dicot seedlings to protect the tender shoot apical meristem during soil emergence. Regulated by many phytohormones, the apical hook has been taken as a model to study the crosstalk between individual signaling pathways. Over recent decades, the roles of different phytohormones and environmental signals in apical hook development have been illustrated. However, key regulators downstream of canonical hormone signaling have rarely been identified via classical genetics screening, possibly due to genetic redundancy and/or lethal mutation. Chemical genetics that utilize small molecules to perturb and elucidate biological processes could provide a complementary strategy to overcome the limitations in classical genetics. In this review, we summarize current progress in hormonal regulation of the apical hook, and previously reported chemical tools that could assist the understanding of this complex developmental process. We also provide insight into novel strategies for chemical screening and target identification, which could possibly lead to discoveries of new regulatory components in apical hook development, or unidentified signaling crosstalk that is overlooked by classical genetics screening.

## 1. Introduction

Unlike motile animals, which can elude danger and stressful conditions, sessile plants are forced to develop a series of adaptive strategies to thrive in an ever-changing environment. The apical hook results from differential cell elongation across the inner and outer side of the upper hypocotyl in dicot seedlings [1,2]. It protects the fragile shoot apical meristem against mechanical stress exerted by the soil during etiolated growth, and is crucial for seedling survival [3,4]. Due to its spontaneous nature, which makes it readily observable, the apical hook has been taken as a model to study the mechanisms underlying differential growth for decades [5]. In natural conditions, two environmental factors, soil compaction and light, dictate the process of apical hook development. Two sets of transcription factors, ETHYLENE INSENSITIVE 3/EIN3-LIKE 1 (EIN3/EIL1) and PHYTOCHROME INTERACTING FACTORs (PIFs), integrate intrinsic hormone and external environmental signals to elaborately regulate apical hook development [5,6,7]. Among them, ethylene surrounds the seedlings when compact soil restricts its diffusion [8] and activates EIN3/EIL1 transcription factors. Therefore, the abundance of EIN3/EIL1 means they could serve as indicators of soil conditions. Additionally, mutants with a constitutive activation of ethylene signaling, such as *ctr1-1,* display an exaggerated hook phenotype [9]. Meanwhile, PIFs are also positive regulators of the apical hook and are rapidly degraded upon light exposure via the PHYTOCHROME B (phy B)-mediated light signaling pathway [6,10]. The degradation of PIFs leads to not only the opening of the apical hook, but also the dark-to-light growth transition of etiolated seedlings [10].

It is well-known that PIFs and EIN3/EIL1 directly bind to the promoter of *HOOKLESS1* (*HLS1*), a key regulatory gene in apical hook development, and activate its transcription [6,7,11]. *hls1-1* with a loss of function in HLS1 displays a complete defect in apical hook formation, which cannot be recovered by ethylene or any other phytohormones [6,11]. Although the *pifq ein3 eil1* sextuple mutant phenocopies the *hls1-1* mutant, it remains unclear whether there exist other regulators downstream of the transcription factors other than *HLS1* [6].

Auxin, due to its polar distribution, directly regulates cell elongation and dictates the differential growth at the apical hook [12,13]. A high concentration of auxin accumulates at the concave side of the apical hook, which activates AUXIN RESPONSE FACTORs (ARFs) to promote the transcription of *D CLADE TYPE 2C PROTEIN PHOSPHATASE* (*PP2C-D1*), a negative regulator of cell elongation, to establish growth asymmetry [14]. Peng et al. demonstrated that HLS1 is necessary for the establishment of polar auxin transport (PAT) after apical hook initiation; however, the relationship between HLS1 and PAT is still unclear [15]. To date, possibly due to genetic redundancy or lethal mutation, only one regulator downstream of HLS1 has been identified. The mutation of *ARF2* could slightly rescue the developmental defect of apical hook in *hls1-1* mutant, indicating that HLS1 might regulate apical hook development via negatively regulating ARF2, although the effect is rather weak [16]. It is noteworthy that Arabidopsis LAZY1 and LAZY1-LIKEs (LZYs) were reported to be involved in the gravitropism of roots and shoots via regulating PAT [17,18]. Despite the mode of actions of LAZY1/LZYs being characterized in root gravitropism [19,20,21], there is still no report on their roles in apical hook development. Hence, it is increasingly interesting to utilize novel approaches to investigate the regulation of PAT by HLS1 and LAZY1/LZYS in apical hook, as well as to uncover their downstream components.

Chemical genetics has emerged as a powerful tool in plant science research as a complementation of mutant screening-based classical genetics in the past two decades [22,23]. Chemical screening is usually much easier to handle than classical genetics screening, due to the absence of mutagens such as EMS (ethylmethylsulfone) [24]. Moreover, chemical inhibitors generally inhibit proteins with similar ligand-binding pockets, hence overcoming the genetic redundancy of the targets [22]. To date, there has been no report on how HLS1 interacts with polar auxin transport, and there remain certain key regulatory components to be discovered. Due to the high genetic redundancy in auxin signaling and transport mechanisms, chemical genetics might greatly complement the discovery of the mysterious components in apical hook development.

## 2. Recent Advances in Hormonal Regulation of the Apical Hook

**Figure 1:**
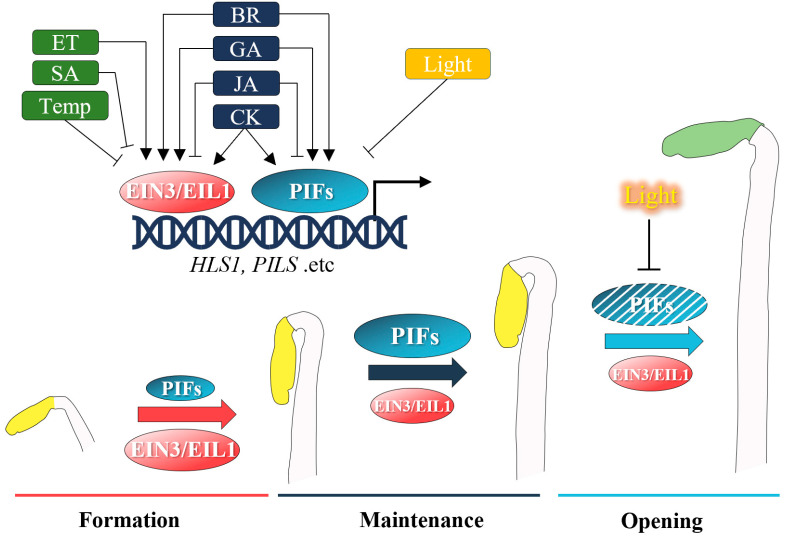
The hormonal regulatory network fine-tuning apical hook development. Multiple intrinsic hormonal signals and extrinsic environmental factors regulate apical hook development via the transcriptional hub formed by EIN3/EIL1 and PIFs. The six transcription factors integrate upstream signals and bind to promoters of genes that are related to apical hook development, such as HLS1, PILs, etc. In the dynamic development process, EIN3/EIL1 play a predominant role during apical hook formation, while PIFs are more crucial during apical hook maintenance in darkness. Light exposure results in the immediate degradation of PIFs, but not EIN3/EIL1; the apical hook undergoes post-light exposure opening because the key players of maintenance, PIFs, are degraded. ET, thylene; SA, Salicylic acid; Temp, high temperature; BR, Brassinosteroid; GA, Gibberellic acid; JA, Jasmonic acid; CK, Cytokinin.

Since the biological function of the apical hook is to protect the fragile cotyledon while seedlings penetrate through soil, two predominant factors that regulate its developmental dynamics are the degree of soil compaction and intensity of light. The transcription factors EIN3/EIL1 and PIFs are responsible for responding to the soil compaction and light, respectively [5,6,7,8]. Moreover, the six transcription factors (EIN3, EIL1, PIF1, PIF3, PIF4, and PIF5) form a signaling hub, which is indispensable for all reported phytohormones that impinge on apical hook development [5] (Figure 1).

NON-EXPRESSER OF PR 1 (NPR1), which is bound and activated by salicylic acid (SA) and responsible for SA-induced transcriptional reprogramming events, suppresses apical hook formation via interacting with EIN3, and inhibits its binding to the *HLS1* promoter [25,26,27]. Meanwhile, jasmonic acid (JA), a hormone that mediates the response to wounding, antagonizes apical hook development via both EIN3 and PIFs. On the one hand, the JA-activated transcription factor MYC2 promotes the expression of *EIN3-BINDING F-BOX PROTEIN 1* (*EBF1*), which encodes an E3-ubiquitin ligase that ubiquitinates EIN3/EIL1 to promote their degradation [28]. On the other hand, MYC2 interacts with both EIN3 and PIF4 to inhibit their transcriptional activation of *HLS1*, thereby antagonizing apical hook development [6,29,30]. Apart from intrinsic phytohormones, environmental stress conditions could also affect apical hook development. A high ambient temperature antagonizes the ethylene-induced exaggeration of apical hook curvature via disrupting the expression of auxin biosynthetic genes *YUCCAs*, which encode rate-limiting enzymes in Trp-dependent auxin biosynthesis, therefore reducing auxin content and attenuating its polar distribution [29].

Meanwhile, positive regulators, including gibberellin Acid (GA), cytokinin (CK), and brassinosteroid (BR), also influence the apical hook via PIFs and theEIN3/EIL1 regulatory hub. GA promotes apical hook development via the de-repression of the DELLA-mediated inhibition of EIN3 as well as PIFs [30,31], which is supported by an exaggerated hook phenotype observed in the *della* quintuple mutant. In comparison, the mode-of-action of cytokinin is more complex. As downstream regulators that are activated by cytokinin via a two-component signaling pathway, B-type ARABIDOPSIS RESPONSE REGULATORs (ARRs) ARR1/10/12 promote the transcription of type-2 *ACC SYNTHETASE* (*ACS*) genes, which encode a key enzyme in the ethylene biosynthesis pathway, and stabilize the ACS proteins from degradation by proteasome [32]. In addition to promoting ethylene biosynthesis, cytokinin also facilitates apical hook maintenance in darkness via the post-transcriptional stabilization of PIF4 and PIF5 transcription factors [33]. BR facilitates apical hook development by activating its master transcription factor BRASSINAZOLE RESISTANT1 (BZR1), which can interact with both EIN3 and PIF4 to synergistically promote the transcription of downstream targets, including *HLS1* [34,35].

In previous studies, the angle of apical hook curvature was taken as a phenotypic output to study hormone signaling crosstalk; however, apical hook development is a dynamic process awaiting thorough understanding [5]. The development of the apical hook could be roughly divided into three phases: formation, maintenance, and opening, which happen in a sequential order at 0–24 h, 24–48 h, and after 48 h post-germination [1,2,13]. It has been demonstrated that cytokinin and ethylene display different modes of action in the dynamic developmental progress of the apical hook, although they both trigger the exaggeration of the hook angle. Cytokinin prolongs apical hook maintenance in darkness in a PIF-dependent and EIN3/EIL1-independent manner, pinpointing the functional divergence of PIFs and EIN3/EIL1 [33]. There exists evidence for the molecular mechanisms underlying the capability of PIFs to promote hook maintenance. First, PIFs inhibit the expression of *PIN-LIKES* (*PILS*), which encode putative auxin carriers and are known to be negative regulators of apical hook maintenance, since their loss-of-function mutants display longer apical hook maintenance [36]. Moreover, PIFs also inhibit the cytokinesis of cells residing on the inner side of the apical hook, further facilitating the maintenance of differential growth [37]. Given the fact that hormones such as cytokinin also affect cytokinesis, it would be interesting to investigate whether this also contributes to certain aspects of hook development, either dependent on or independent of PIFs. Despite our understanding of the dynamic regulation of the apical hook by cytokinin and ethylene, how other phytohormones and environmental cues control the different phases of apical hook development remains largely unexplored.

Furthermore, it is worth noting that different phytohormones might exert their effects in regulating apical hook development via distinct physiological roles. For instance, although both ethylene and gibberellin promote apical hook development, they display opposite effects on the elongation of hypocotyl [30,38]. Since the establishment of the apical hook results from differential elongation across the inner and outer cells [13], it is intriguing to observe how these hormones regulate apical hook development at cellular resolution.

## 3. Existing Chemical Tools That Could Help Us Understand Apical Hook Development

Being the first discovered phytohormone, the actions and dynamics of auxin have been extensively investigated over recent decades [39]. Chemical biologists have also participated actively in this field and have developed a set of chemical tools that have provided a profound understanding of auxin actions [40,41]. Given the importance of the phytohormone auxin, which directly impinges the differential cell elongation across the inner and outer sides of the apical hook, many chemical regulators of auxin signaling, as well as polar transport, could significantly affect apical hook development. Here, we summarize the chemical regulators that could facilitate the research of the apical hook (Table 1).

### 3.1. Chemicals Regulating Auxin Biosynthesis and Metabolism

**Figure 2:**
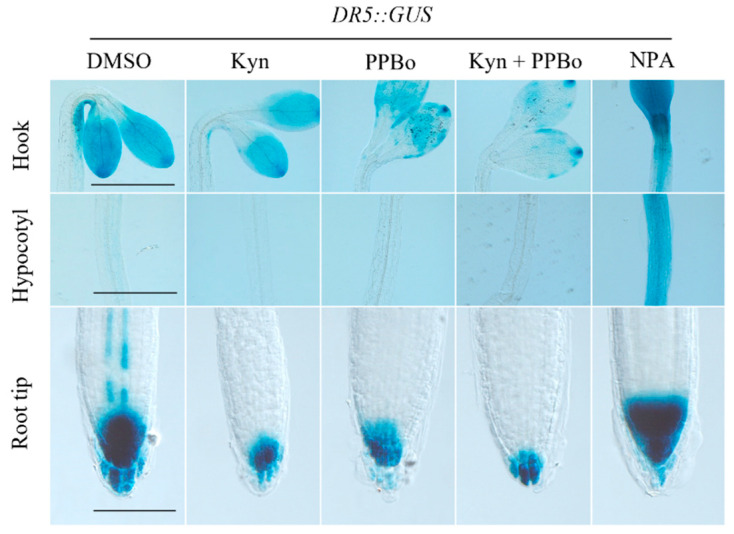
Effects of auxin synthesis and transport regulators on apical hook development and auxin signaling. GUS staining of 3.5-day-old etiolated Arabidopsis seedlings grown on 1/2 MS medium with or without the addition of DMSO, 5 mM Kyn, 5 mM PPBo, 5 mM Kyn + 5 mM PPBo, or 5 mM NPA. The GUS reported was driven by a synthetic, auxin-responsive DR5 promoter in Columbia-0 (Col-0) background and stained for 8 h in darkness at 37 °C. Scale bar = 1000 mm in hook and hypocotyl; scale bar = 200 mm in root tip.

Chemical regulators influencing auxin biosynthesis usually target the tryptophan (Trp)-dependent auxin synthetic pathway, since it is the predominant route [42]. L-kynurenine (Kyn), a structural mimic of Trp, has been reported to inhibit the enzymatic activities of TRYPTOPHAN AMINOTRANSFERASE OF ARABIDOPSIS 1/TRYPTOPHAN AMINOTRANSFERASE RELATEDs (TAA1/TARs) that converts tryptophan into indole-3-pyruvic acid [43]. Kyn significantly inhibits primary root length in green seedlings and the auxin signaling marker *DR5::GUS,* staining signals at the inner side of the apical hook; however, its effects on apical hook curvature are rather weak [43] (Figure 2). This implies that a lower degree of auxin asymmetry is sufficient to mediate the differential growth at the apical hook, although its further effects on later stages of apical hook development, including maintenance and opening, remain to be further studied. In comparison, p-phenoxyphenyl boronic acid (PPBo), a YUCCA flavin mono-oxygenase inhibitor, displayed more potent effects, which could be explained by a canonical notion that YUCCAs are rate-limiting steps in auxin biosynthesis [44] (Figure 2). By using KOK2099 as a chemical tool, a recent report demonstrated that indole-3-pyruvic acid (IPyA), as a major intermediate product of IAA biosynthesis, can competitively inhibit TAA1 and be reversely converted to Trp by TAA1 [45], which deepens our understanding of IAA biosynthesis mechanism. Since chemical regulators usually inhibit several homologous target proteins, and the dose and application time window can be flexibly regulated, a combination of Kyn and PPBo could possibly be utilized to explain how local auxin biosynthesis fine-tunes the dynamic process of apical hook development. There are also many other regulators (Table 1) that target TAA1 or YUCCA that can facilitate the regulation of endogenous auxin levels [46,47,48,49,50].

**Figure 3:**
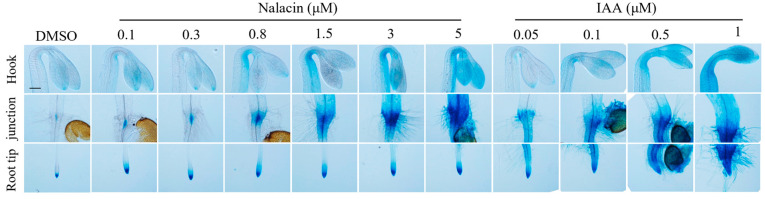
Effects of nalacin and IAA on apical hook development and auxin signaling. GUS staining of 3.5-day-old etiolated Arabidopsis seedlings grown on 1/2 MS medium with or without the addition of DMSO, nalacin, or IAA, with concentrations indicated in the figure. The GUS reported was driven by a synthetic, auxin-responsive DR5 promoter in Columbia-0 (Col-0) background and stained for 4 h in darkness at 37 °C. Scale bar = 200 mm.

The major auxin inactivation pathways in Arabidopsis are catalyzed by two families of enzymes. DIOXYGENASE FOR AUXIN OXIDATION 1 (DAO1) and its homolog DAO2 are 2-oxoglutarate and Fe(II)-dependent oxygenases that oxidize IAA into oxIAA, an inactive form [51,52]. In parallel, another family of acyl acid amido synthetases GRECHEN HAGEN 3 (GH3) catalyzes reversible auxin conjugation to amino acids, resulting in their inactivation [53,54]. The GH3 protein family can be generally divided into three subfamilies: groups I, II, and III of GH3 conjugates amino acid to salicylic acid (SA), indole-3-acetic acid (IAA), and jasmonic acid (JA), respectively [53,55]. To date, three small-molecule inhibitors targeting GH3 enzymes have been developed. Adenosine-5′-[2-(1H-indol-3-yl) ethyl] phosphate (AIEP), which structurally mimics adenylated IAA intermediate during amino acid conjugation reaction, competitively inhibits the in vitro enzymatic activities of GH3.1 and GH3.6 in the Grape [56]. Kakeimide (KKI) also displays competitive inhibition on auxin-conjugating GH3s and biological activities on root growth and development [57]. Additionally, high-dose KKI treatment phenocopies the septuple mutant of group II GH3s [57]. Conversely, another reported GH3 inhibitor, nalacin, can elevate local auxin concentration via inhibiting auxin-conjugating group II GH3s [58]. Exogenous treatment with IAA reduces the angle of curvature, possibly by hindering the asymmetrical distribution of auxin signal across the apical hook (Figure 3). In contrast, nalacin treatment can promote the formation of a fully closed apical hook together with a more enhanced auxin asymmetry (Figure 3). These results indicate that the increase in endogenous auxin level does not jeopardize apical hook development, while the exogenous application of auxin breaks the asymmetrical distribution and leads to defects in hook formation. Moreover, the accumulation of the auxin signal at the inner side of the apical hook upon nalacin treatment indicates a potential role of GH3-mediated auxin conjugation in apical hook development. It would be exciting to utilize nalacin, together with the aforementioned tools, to study the role of auxin metabolism in the formation, maintenance, and post-light exposure opening processes of the apical hook, which awaits further investigation.

### 3.2. Chemicals Regulating Auxin Transport and Signal

The polar distribution of auxin is tightly controlled by influx carriers that transport auxin into the cells, and efflux carriers that function oppositely, and the transporter proteins are also regulated at multiple levels including reversible protein phosphorylation and proteosomal degradation [59,60,61,62]. 1-Naphthoxyacetic acid (1-NOA) has been reported to inhibit auxin influx into tobacco BY-2 cells [63]. Moreover, 1-NOA-treated Arabidopsis seedlings phenocopies *aux1* mutant, displaying disordered root gravitropic bending [64]. Taken together, 1-NOA possibly blocks auxin influx via targeting the AUX1/LAX auxin influx carriers. Compared to auxin influx, the mechanisms of auxin efflux via PIN-FORMED efflux carriers have been extensively investigated, and abundant chemical regulators have been reported to interfere with this process [23]. Among them, Naphthylphthalamic acid (NPA), which associates with PINs and inhibit their functions, has been thoroughly studied and widely used in the study of auxin actions [65,66]. Very recently, the structure of PIN8, with or without NPA binding, has been resolved with single-particle cryo-EM; this further clarified the mode-of-actions of PINs in auxin transport and could possibly guide the design of more potent regulators [67]. Etiolated Arabidopsis seedlings treated with 10 mM NPA or 1-NOA fully abolished apical hook formation (Figure 2). However, NPA treatment strongly induced the accumulation of auxin at the upper hypocotyl, the site of apical hook formation, indicating the active auxin efflux event that happens at this region (Figure 2). In contrast, although 1-NOA phenocopies NPA treatment, it does not significantly promote the auxin accumulation in the hypocotyl. Instead, 1-NOA resulted in the accumulation of auxin within the cotyledon, where auxin was predominantly synthesized, possibly due to the inability of transporting auxin to other parts of the seedling (Figure 2). Through a screening of the auxin analog that affects PIN trafficking, pinstatic acid (PISA) was identified [68]. PISA can interrupt the gravitropism of the root, possibly via influencing the accumulation and internalization of PIN, but not activating TIR1/AFB-mediated signaling. It would be interesting to investigate the effects of PISA in apical hook development. Besides transport, uxin perception is also a key regulating point for apical hook development. Several chemical regulators that target the auxin receptor have been reported, such as auxinole as an antagonist [69], fluorescent auxin analogs [70], and selective agonists for specific subsets of AUX/IAA [71], among which auxinole was applied to dissect the developmental processes of the apical hook [15]. In addition, an orthogonal auxin–TIR1 receptor pair (convex IAA–concave TIR1) has been developed [72], providing a strategy for the precise manipulation of auxin signal. These receptor regulators and orthogonal pair could serve as chemical toolkits for the study of apical hook development.

### 3.3. Other Chemicals That Regulate the Apical Hook

Small-molecule regulators of the apical hook that do not directly correlate with auxin actions are rather scarce. Upstream of the polar auxin transport events, functional microtubule arrays are indispensable for the correct distribution of auxin transporters, and guarantee the establishment of auxin asymmetry [73,74]. The application of 50 mM oryzalin completely abolished the normal apical hook formation in Col-0, and a lower concentration (200 nM) could revert the positive contribution of the external mechanical constraint on apical hook development in the *ktn1-5* mutant [15,73], hinting at a fundamental role of functional microtubule arrays in hook development. Moreover, a previous chemical screening using the co-treatment of compounds with ACC, a precursor of ethylene, identified several interesting compounds that either promote or inhibit the apical hook [75]. Among them, 6,825,783 inhibits both apical hook formation and ethylene signaling, while restoring the shortening of root induced by ethylene, hinting that it could possibly be a regulator of ethylene signaling. Notably, another compound, 7545271, facilitates the ethylene-induced exaggeration of the apical hook, while antagonizing ethylene signaling. Given the positive role of ethylene in apical hook development, the co-occurrence of the two events seems intriguing and demonstrates the complexity of the regulatory network of the apical hook. EH-1, a pyrazole derivative, was identified to trigger apical hook exaggeration in an ethylene-independent manner [76]. However, to date, none of these compounds has a clear mode-of-action or is known to target proteins in Arabidopsis. In addition, researchers also designed a screening system that aimed to identify apical hook promoters that are independent of ethylene signaling. Using *ein3 eil1* ethylene-insensitive mutant seeds as the substrate, two small-molecule compounds, Apical Hook Inducer 1 (AHI1) and AHI2, were identified. Among them, AHI1 was identified to be a cytokinin precursor kinetin riboside, which functions after being metabolized into a free base active form [33]. Meanwhile, AHI2 directly promotes PIN-dependent polar auxin transport via promoting PIN3 accumulation, in addition to the intracellular trafficking of PIN2 (unpublished). Notably, AHI2 not only promotes the apical hook but also other auxin transport-related phenotypes, including the gravitropic bending of hypocotyl and root, as well as adventitious root formation, suggesting that AHI2 might be a positive regulator of polar auxin transport and the relevant responses. Further studies on the modes of action of the above-mentioned chemical regulators could assist in deepening our understanding of apical hook development.

## 4. Developing Chemical Tools with Novel Targets to Further Delineate Apical Hook

Chemical biology is a powerful tool for drug discovery. Chemical genetics screening is one of the major strategies for developing plant growth regulation [23]. The apical hook forms stably in a short time-window (3–4 days in darkness) and the etiolated seedings are a suitable size to fit for 96-well plates, making them an excellent model for high-throughput phenotype-directed screening (PDS) for bioactive chemicals. Since apical hook development is orchestrated by multiple signals [5], the PDS should be performed with mutants or marker lines, rather than wild-type Col-0, to make the screen more specific for discovering novel targets.

A target search of small-molecule regulators is a key step for chemical genetics studies and has been a time-consuming step and bottleneck for a long time. Although many available regulators in apical hook development have been reported, the targets and modes of action are yet to be determined. Previously described methods mostly concerned the study of drug actions in animal cells, while less attention has been paid to their applications in plant chemical genetics [77]. Owing to recent progress in both analytic techniques, the targets of several chemical probes have been determined by proteome-wide cellular thermal shift assay (CETSA) [78,79]. High-resolution mass spectrometry-based profiling of the proteome could reveal proteins whose stability has changed upon chemical treatment [77]. In Arabidopsis, CETSA was performed with intact PSB-D suspension cell cultures or cell lysates [80,81]. Intact cells with functional signaling pathways allow the investigation of not only the target protein alone, but also proteins that are modified downstream of the direct target. Meanwhile, cell lysate is more powerful when the direct target is to be spotted [80,81].

Protein–ligand docking has been proven to be a powerful approach towards the understanding of protein–small molecule interactions, and the discovery of novel regulators of a specific protein of interest [82,83,84,85]. Due to the development of artificial intelligence and big data science, AlphaFold has predicted over 200 million structures of various organisms, including Arabidopsis and rice, which are released to a database with free access [86]. This revolutionary advance not only makes it easier to obtain highly confident predicted protein structure of interest to perform high-throughput virtual screening, but also provides the possibility of reverse docking (RevDock) with a single small-molecule ligand to proteome-wide pockets in one or multiple organisms. With a refined algorithm, a novel protein–ligand scoring function, OnionNet-SFCT, was developed to assist the target search of ligands [87]. This artificial intelligence (AI) drug discovery and design (AIDD) strategy displayed satisfactory precision, for a well-established plant hormone ABA, which is identified by 14 receptors *in planta*, 4 of which could be found in the top 10 interacting proteins, and 8 of which could be found in the top 100 proteins [87]. Taken together, although both CETSA and RevDock possess a certain degree of error rate, we can suppose that the combination of these two methods would be promising in improving the drug target identification. In other words, if a protein could be identified in CETSA-MS analysis, and be ranked front in RevDock, it would be worthwhile to examine if the protein could indeed interact with the ligand.

## 5. Conclusions and Future Perspectives

To date, a rather complex hormone framework regulating apical hook development has been illustrated, and the role of individual signaling pathways has been extensively studied. Nevertheless, it is noteworthy that epigenetic regulation is important in many aspects of plant growth and development, and it shows plasticity in response to environmental cues [74] and interplays with phytohormones, regulating somatic embryogenesis, seedling development, flowering, and developmental plasticity at multi-layered levels [88,89,90,91,92]. In considering that HLS1, a key factor in apical hook development, is a putative histone acetyltransferase, the relationship between apical hook development and epigenetics, including DNA methylation, histone modification, and chromatin remodeling, should be one of the major open questions being investigated. To gain a better understanding of the physiological significance of the apical hook, together with the regulatory mechanisms behind it, the following issues need to be addressed: (1) Resolve the molecular mechanism of stress-induced ethylene biosynthesis, which is crucial for seedlings to sense the compaction of soil when buried underground; (2) identify key regulators downstream of HOOKLESS1; (3) clarify the relationship between epigenetics and apical hook development; and (4) utilize transcriptomic analysis and genetics to identify key genes that are involved in the dynamic process of apical hook formation, maintenance, and opening. The ultimate goal of these studies is to provide a holistic view of the molecular landscape governing the spatial-temporal development of the apical hook.

Chemical genetics overcomes the genetic redundancy and lethality of mutations, and therefore provides a great opportunity to address the remaining questions by overcoming the bottleneck of classical genetics. With emerging techniques to identify target proteins of small-molecule regulators, it is increasingly promising to reveal the mechanisms underlying apical hook development via studies towards chemical tools. More importantly, the core mechanism for apical hook development is asymmetric growth that determines many aspects of plant tropism responses. The mechanism dissection of apical hook development with chemical tools is expected to deepen our insights into more general and fundamental mechanisms, and provide us with chemical tools in both basic research and agricultural applications.

## Figures and Tables

**Table 1:** Available chemicals for dissecting apical hook development.

	Full Name	Description	Reference	CAS NO.
Auxin biosynthesis	L-Kynurenine (Kyn)	TAA1/TAR2 inhibitor	[43]	2922-83-0
	Pyruvamine2031	OSTAR1 inhibitor	[47]	N.A
	p-Phenoxyphenyl boronic acid (PPBo)	YUCCAs inhibitor	[44]	51067-38-0
	Yucasin	YUCCAs inhibitor	[48]	26028-65-9
	Yucasin DF	YUCCAs inhibitor	[49]	443797-96-4
	Ponalrestat (PRT)	YUCCAs inhibitor	[50]	72702-95-5
Auxin metabolism	Adenosine-5′-[2-(1H-indol-3-yl)ethyl]phosphate (AIEP)	GH3 inhibitor	[56]	260430-02-2
	Kakeimide (KKI)	GH3 inhibitor	[57]	N.A
	Nalacin	GH3 inhibitor	[58]	1019105-44-2
Auxin transport and signaling	1-naphthoxyacetic acid (1-NOA)	Putative AUX1/LAXs inhibitor	[63,64]	2976-75-2
	2-naphthoxyacetic acid (2-NOA)	Putative AUX1/LAXs inhibitor	[63,64]	120-23-0
	Naphthylphthalamic acid (NPA)	PINs inhibitor	[65,66]	132-66-1
	4-ethoxyphenylacetic acid (PISA)	Auxin transport promoter	[68]	132-66-1
	Auxinole	Auxin receptor agonist	[69]	86445-22-9
	NBD-IAA	Fluorescent auxin analog	[70]	N.A
	RN1-4	Selective auxin agonists	[71]	N.A
	cvxIAA-ccvTIR1 pair	Engineered IAA-TIR1 pair	[72]	N.A
Other regulators	Oryzalin	Apical hook suppressor	[16,73]	19044-88-3
	6825783	Apical hook suppressor	[75]	N.A
	7545271	Apical hook promoter	[75]	N.A
	Apical Hook Inducer 1 (Kinetin Riboside)	Apical hook promoter	[33]	4338-47-0
	Apical Hook Inducer 2	Apical hook promoter	unpublished	N.A

## Data Availability

Not applicable.
